# Distinguishing recent dispersal from historical genetic connectivity in the coastal California gnatcatcher

**DOI:** 10.1038/s41598-018-37712-2

**Published:** 2019-02-04

**Authors:** Amy G. Vandergast, Barbara E. Kus, Kristine L. Preston, Kelly R. Barr

**Affiliations:** 1 U. S. Geological Survey, Western Ecological Research Center, Sacramento, CA USA; 2 San Diego Management and Monitoring Program, San Diego, CA USA; 3 Present Address: Center for Tropical Research, Institute for the Environment and Sustainability, University of California-Los Angeles, Los Angeles, CA USA

## Abstract

Habitat loss and fragmentation are primary threats to biodiversity worldwide. We studied the impacts of habitat loss and fragmentation on genetic connectivity and diversity among local aggregations of the California gnatcatcher (*Polioptila californica californica*) across its U.S. range. With a dataset of 268 individuals genotyped at 19 microsatellite loci, we analyzed genetic structure across the range using clustering analyses, exact tests for population differentiation, and a pedigree analysis to examine the spatial distribution of first-order relatives throughout the study area. In addition, we developed a habitat suitability model and related percent suitable habitat to genetic diversity indices within aggregations at two spatial scales. We detected a single genetic cluster across the range, with weak genetic structure among recently geographically isolated aggregations in the northern part of the range. The pedigree analysis detected closely related individuals across disparate aggregations and across large geographic distances in the majority of the sampled range, demonstrating that recent long-distance dispersal has occurred within this species. Genetic diversity was independent of suitable habitat at a local 5-km scale, but increased in a non-linear fashion with habitat availability at a broader, 30-km scale. Diversity declined steeply when suitable habitat within 30-km fell below 10%. Together, our results suggest that California gnatcatchers retain genetic connectivity across the majority of the current distribution of coastal sage scrub fragments, with the exception of some outlying aggregations. Connectivity may help support long-term persistence under current conservation and management strategies. However, emerging structure among more remote aggregations and associations between available habitat and genetic diversity also suggest that continued loss of habitat could threaten diversity and connectivity in the future.

## Introduction

Habitat loss and fragmentation are key factors that influence population loss and species extinction^1^. Persistence of rare species in fragmented systems can depend on the extent to which local populations are linked both demographically and genetically by dispersal. Particularly for species with small, non-migratory populations, connectivity can be imperative for the maintenance of genetic diversity through gene flow (which is maintained by movement and successful reproduction), reestablishment after local extinctions, and avoidance of inbreeding depression^2,3^. Because habitat fragmentation can impede the dispersal capabilities of even volant species^4,5^, the identification of these restrictions to gene flow can be essential for long-term species conservation planning.

Dispersal can be directly observed, or estimated indirectly. For example, banding and resighting studies are commonly used to estimate dispersal in small songbirds. However, study area size limitations in resighting or recapture surveys can systematically bias the obtained dispersal distributions, and tend to miss or underestimate long distance dispersal events^6^. While radio or satellite tracking methods can alleviate some of these biases, transmitter and battery size limitations may make such methods untenable for small species^7^. Alternatively, estimates of population genetic structure have been used to assess population connectivity and indirectly estimate long distance dispersal^8,9^. However, in historically large or well-connected populations, the magnitude and detectability of recent changes to genetic structure can reflect other factors besides movement, such as population size, generation times, historical connectivity and the variability and mutation rates in the chosen genetic markers^10–12^. For these reasons, it can be difficult to determine whether a lack of detectable genetic differentiation among fragmented populations reflects high ongoing levels of dispersal and gene flow, or is a retained signal of historically high gene flow that does not yet reflect current levels of dispersal and gene flow^13–15^.

Previous studies have used several techniques to help distinguish between historical and emerging contemporary genetic patterns. Some have assessed differences in correlations between historical versus current landscape connectivity patterns and genetic differentiation^16,17^. Others incorporate temporal sampling to estimate population genetic structure before and after disturbance^18,19^, or compare levels of genetic connectivity in fragmented versus contiguous habitat^4,20^, or compare among species with different dispersal abilities^8,21^. Multilocus genotypes can also be used to statistically estimate recent migration rates^22^. However, where there is no detectable signal of genetic subdivision, these methods are of limited use^23^. In cases with no apparent genetic subdivision, an alternative method to distinguish between high ongoing gene flow versus a retained pattern of historically high gene flow is to estimate kinship among sampled individuals from highly variable genetic markers in a pedigree analysis^24^. This type of analysis can provide context about recent movement and gene flow by examining the geographic distribution of genetically identified first order relatives (full siblings or parents and offspring)^25,26^.

We examined population genetic structure and estimated relationships within a federally threatened songbird, the coastal California gnatcatcher (*Polioptila californica californica;* hereafter gnatcatcher). The gnatcatcher is a resident, non-migratory songbird with a range extending from Ventura County, California, to northern Baja California, Mexico, and is considered a flagship species for coastal sage scrub conservation in southern California^27^. It nests and forages almost exclusively in native coastal sage scrub habitat, an estimated 80–90% of which has been lost since European settlement^28^.

The number of gnatcatcher pairs declined dramatically throughout southern California in the 1980s and 1990s, and as a consequence, the subspecies was listed in 1993 as federally threatened under the Endangered Species Act^29^. The listing status was recently retained in a twelve month finding^30^. The gnatcatcher is also protected in several southern California regional conservation plans (https://www.fws.gov/carlsbad/HCPs.html). Current estimates of the regional population size range between 1000 to 2000 pairs^30^. Previous behavioral studies of gnatcatchers in southern California found that birds were resident in breeding territories (average 8.1 ha) and non-breeding home ranges (12.4 ha)^31^, and that longer distance dispersal occurred mostly by fledglings dispersing away from natal territories^32^. Information on dispersal distance is limited, but a few mark-resighting efforts observed movements up to 14 km. Limitations to dispersal could make remaining aggregations vulnerable to impacts from habitat fragmentation. Therefore, efforts to determine population status and manage for persistence could be assisted by a better understanding of population connectivity across southern California. The goals of our study were to determine whether habitat fragmentation in the U.S. portion of the range has led to population genetic subdivision, and to estimate the geographic extent of recent dispersal. Results can inform future recovery management and monitoring efforts throughout southern California.

## Results

### Data Quality

**Figure 1:**
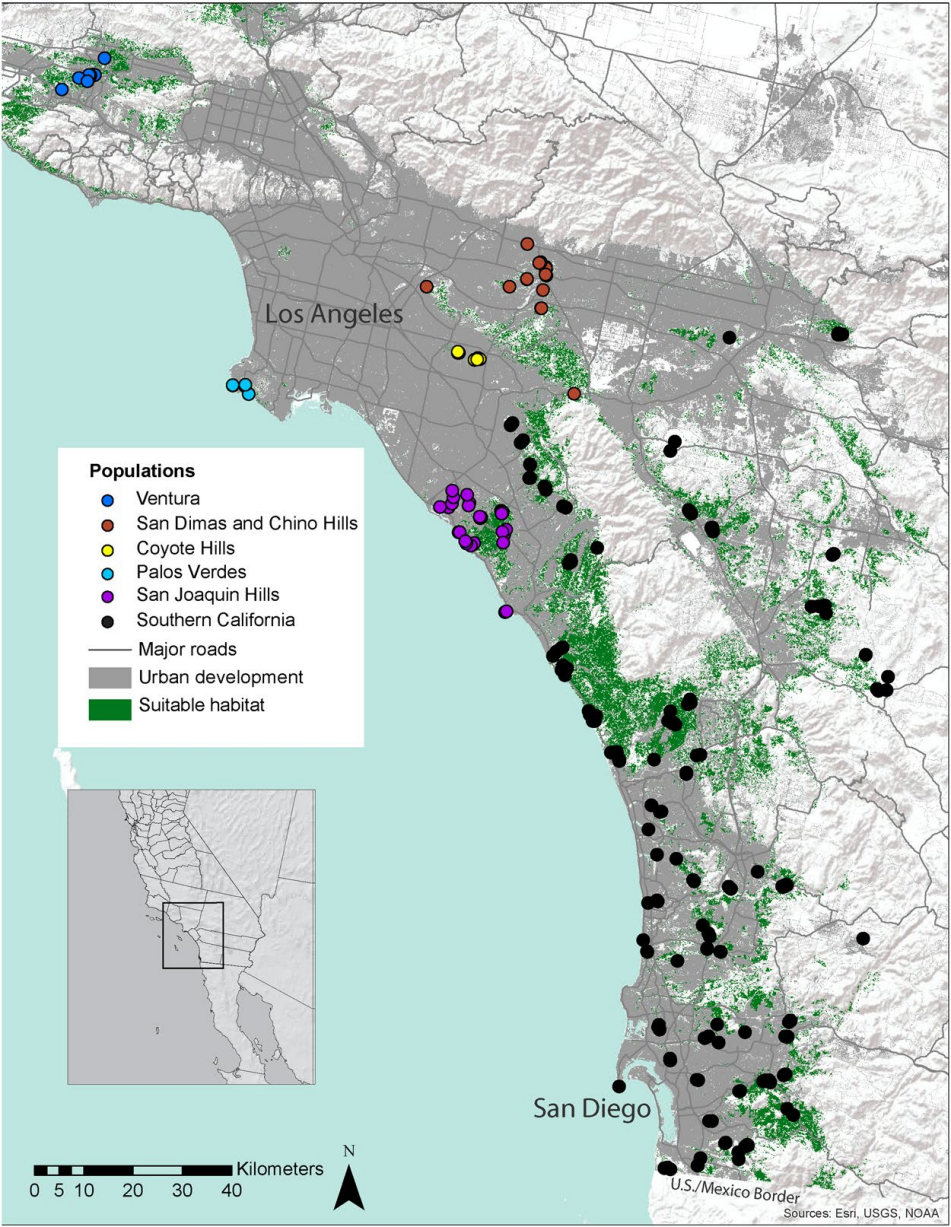
Individual sampling locations of California gnatcatchers throughout the study area. Insert shows southern California study area. Points are colored by populations assigned using the Waples and Gaggiotti^33^ method of exact tests for differences among aggregations. Aggregations without statistically significant allele frequency differences are grouped together. Suitable habitat was calculated from the habitat model and defined as grid cells with a habitat suitability index ≥0.5. The urban centers of the cities of Los Angeles and San Diego are labeled.

We genotyped 268 gnatcatchers sampled throughout their U.S. range at 26 variable microsatellite loci (Fig. 1; Supplementary Table S1). Seven of 26 loci were removed from analyses because of amplification inconsistencies or for not exhibiting Hardy-Weinberg equilibrium across multiple collection localities. In the 19 loci retained for subsequent analyses, we estimated <0.1% missing data, low error rate (<0.1%), and found no linkage disequilibrium among loci or aggregations.

### Population Structure

Three different clustering methods provided no evidence for genetic structure in the study area in analyses on the full dataset (STRUCTURE, GENELAND, and PCA; Supplementary Figs S1–S3) and a subset that excluded close relatives (removing 31 individuals and retaining 237; results not shown). All clustering methods indicated that the full range of gnatcatchers in the U.S. likely forms a single genetic cluster with little differentiation (Supplementary Figs S1–S3). Using exact tests for population differentiation^33^ to further test for population subdivision among 18 regional aggregations, we found evidence for population differentiation at the more northern extent of the study area. This method can be more sensitive at detecting recently formed or weaker levels of genetic structure than clustering algorithms^33^, and so results could suggest subtle or emerging restrictions to gene flow in response to habitat loss and fragmentation. These results varied between analyses on the full dataset and the dataset excluding close relatives. Because the effects of close relatives on genetic structure analyses is a subject of debate^34^, we present both results here. Using the full dataset, five northern and fragmented aggregations were supported as distinguishable populations based on allele frequency differences (Ventura, Palos Verdes, Chino Hills, Coyote Hills and San Joaquin Hills), while all other collection locations from the eastern Los Angeles Basin through southern San Diego County formed a single population (Fig. 1; Supplementary Table S2). However, only Ventura remained distinguishable when one of each first order relative pairs was excluded. Pairwise F_ST_ calculated among aggregations ranged from 0 to 0.089 (Supplementary Table S3) and there was a significant pattern of isolation by distance across the range (Mantel Test Z = 478416.461, r = 0.608, one-sided *p* ≤ 0.001 from 1000 randomizations).

### Pedigree analysis and distances among relatives

Trace plots of COLONY runs on empirical data showed convergence and stationarity among three separate runs (log likelihood scores: −30164.90, −30154.71, −30129.39). Thirty-nine putative first order relative pairs were recovered with a probability > 0.9. Thirty-four of these were consistently recovered in all three runs and retained for distance analysis (Table 1). Simulation results under the full likelihood model suggested our empirical data should have reasonably high assignment accuracy for first order relationships. The highest mis-assignment rates were for true full-siblings falsely classified as non-siblings (12.61%), whereas only 0.0001% of non-siblings were falsely classified as full-siblings. For parent offspring classifications, 1.47% of true parents were falsely excluded as parents and 1.64% of non-parents were falsely included as parents. Together these mis-assignment rates suggest that assigned sibling and parental relationships are likely to reflect true relationships, but errors in classifying true siblings as unrelated individuals may be more frequent.

**Table 1:** Thirty-four first order relative dyads supported with P > 0.90 in three COLONY runs.

First order relative pairs	Prob	Euclidean distance (m)	LCP distance (m)	Individual 1 band number	Region 1	Individual 2 band number	Region 2
1	0.972	66411	93387	230059903	Redlands	268015424	San Joaquin Hills
2	1	309	309	230059911	San Pasqual	230059913	San Pasqual
3	1	357	357	230059912	San Pasqual	230059910	San Pasqual
4	1	9541	10,806	230059930	Lakeside	267026817	Mission Trails
5	1	3399	3673	230059931	Lakeside	230059933	Lakeside
6	0.994	917	917	233051492	San Dimas - Chino Hills	267017523	San Dimas - Chino Hills
7	0.922	35978	57491	233051494	San Dimas - Chino Hills	267026857	Santa Ana Mountains
8	1	316	316	233051497	San Dimas - Chino Hills	233051499	San Dimas - Chino Hills
9	1	4525	5220	267017516	Ventura	267017518	Ventura
10	1	5400	8443	267017520	Ventura	267017521	Ventura
11	1	133332	195261	267017527	Otay - Jamul	268015427	Northwestern Riverside
12	0.984	117179	213881	267017529	Otay - Jamul	267026851	Southwestern Riverside
**13**	**1**	**596**	**596**	**267017531**	**San Joaquin Hills**	**267017535**	**San Joaquin Hills**
**14**	**1**	**596**	**596**	**267017532**	**San Joaquin Hills**	**267017535**	**San Joaquin Hills**
15	1	7769	9043	268016144	San Joaquin Hills	267017536	San Joaquin Hills
16	1	25519	30316	267017539	San Joaquin Hills	268016129	San Joaquin Hills
17	0.99	29401	45545	267017543	Northwest San Diego	267026831	Mission Trails
18	0.998	59704	81908	267017544	Northwest San Diego	267026865	Otay - Jamul
19	1	2459	3259	267017670	Palos Verdes	267026809	Palos Verdes
**20**	**1**	**685**	**685**	**267017672**	**Sweetwater**	**267017674**	**Sweetwater**
**21**	**1**	**1163**	**1163**	**267017672**	**Sweetwater**	**267017675**	**Sweetwater**
**22**	**1**	**526**	**526**	**267017674**	**Sweetwater**	**267017675**	**Sweetwater**
23	0.914	93004	148162	267017677	Otay - Jamul	268016106	South Camp Pendleton
24	0.995	53046	84701	267026807	San Dimas - Chino Hills	268016164	Santa Ana Mountains
25	0.977	64453	121612	267026854	Southwestern Riverside	268016143	San Joaquin Hills
26	1	19162	23187	267026863	Cardiff - Los Penasquitos	230059911	San Pasqual
27	0.967	113809	155613	268015401	Sweetwater	268015425	San Joaquin Hills
28	0.961	132113	184969	268015414	Sweetwater	268016152	Santa Ana Mountains
29	0.999	26042	27902	268016105	South Camp Pendleton	268016124	South Camp Pendleton
30	1	2401	2809	268016112	South Camp Pendleton	268016118	South Camp Pendleton
31	1	3614	5158	268016128	San Joaquin Hills	267017536	San Joaquin Hills
32	1	4125	4682	268016130	San Joaquin Hills	268016131	San Joaquin Hills
**33**	**1**	**118**	**118**	**268016157**	**Coyote Hills**	**268016158**	**Coyote Hills**
**34**	**1**	**91**	**91**	**268016159**	**Coyote Hills**	**268016158**	**Coyote Hills**
**mean**		**29943**	**44785**				
**median**		**4963**	**6832**				
**max**		**133332**	**213881**				
**min**		**91**	**91**				

Table includes the probability of assignment, the Euclidean and Least Cost habitat distances between captures, band or identification number, and regional aggregations where each individual was captured. Bolded pairs contain at least one relative in common and likely represent a single family group.

**Figure 2:**
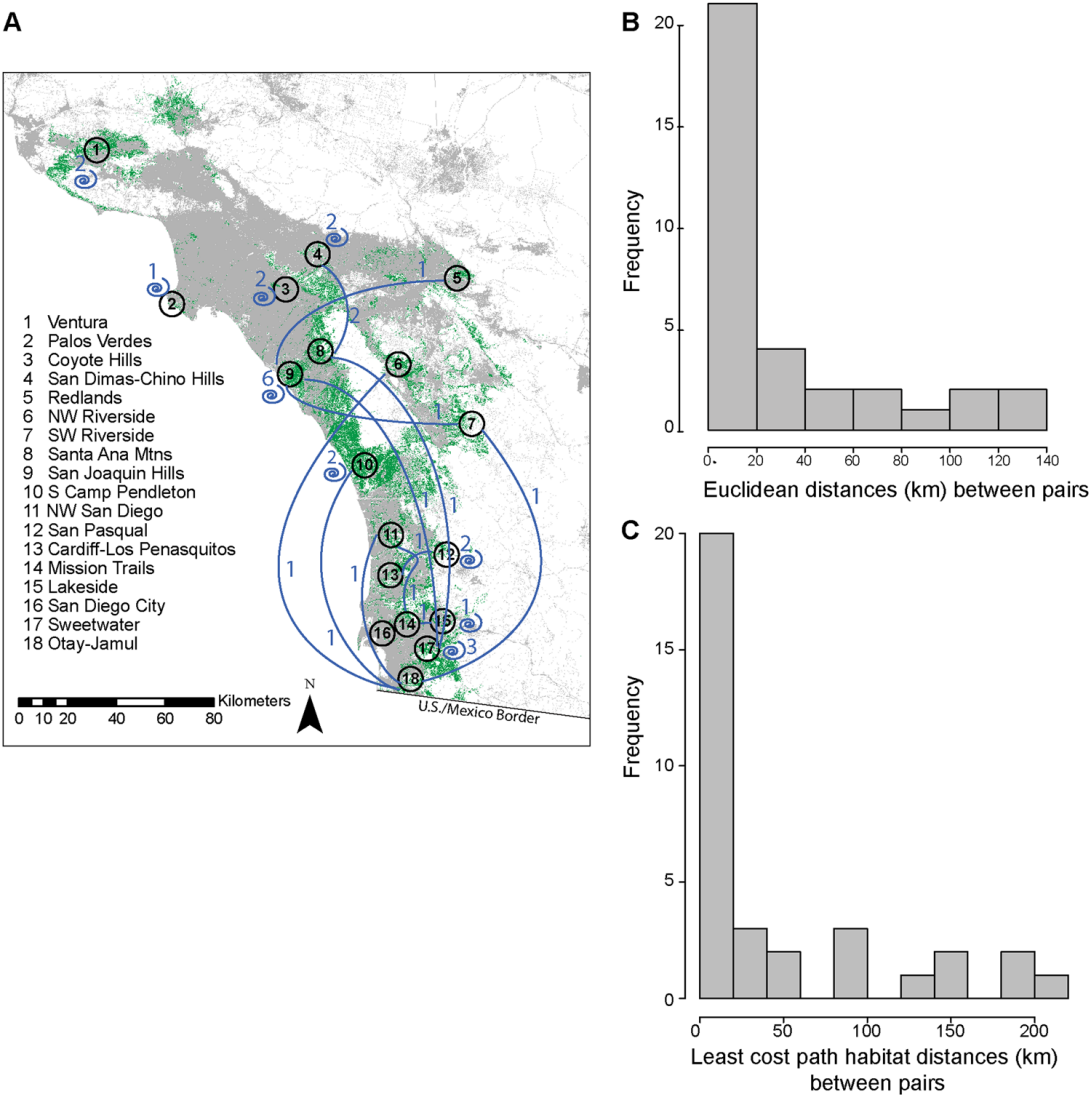
(**A**) Mean center points for 18 regional aggregations overlaid on suitable habitat (green). Blue lines represent connections between regional aggregations based on first order relative pairs. Numbers indicate the number of pairs with individuals shared between aggregations. Spirals and corresponding numbers indicate first order relative pairs with both members found within the same aggregation. (**B**,**C**) Histograms of Euclidean and least cost path distances between relatives. Most distances were under 20 km.

Thirteen of 34 relative pairs were detected across different aggregations (Fig. 2A). The Euclidean geographic distances between capture locations of first order relatives averaged 29.9 km (median 4.9 km) and ranged from 91 m to 133 km (Fig. 2B). Least cost path distances through habitat predicted with a California gnatcatcher habitat suitability model were longer than Euclidean distances, averaging 44.7 km (median 6.8 km) and ranging from 91 m to 214 km (Fig. 2C).

### Effective Population Size and Genetic Diversity

The effective population size estimates for the entire cluster exceeded 1,000 (Table 2). At the level of aggregations, we detected variable genetic diversity levels across the range, with the highest in the southernmost portion of the study area and the lowest in the north, while relatedness (r)^35^ was highest in the northernmost portion of the study area and lowest in the south (Table 2).

**Table 2:** Genetic diversity indices (*H*_o_, *H*_e_, *A*_r_), inbreeding coefficient (F), relatedness (*r*) for regional aggregations and the entire cluster.

		N	*H*_o_	*H*_e_	F	*r*	*A*_r_	N_e_
Entire Cluster		268	0.733	0.785	0.068	−0.004	4.99	1025.8 (669.8–2049)
								2139 (1694–2964)
**Regional Aggregations**								**% suitable habitat in 30-km buffer**
1*	Ventura	10	0.747	0.684	−0.101	0.153	4.13	9.15
2*	Palos Verdes	5	0.705	0.655	−0.089	0.130	4.05	2.94
3*	Coyote Hills	10	0.768	0.713	−0.076	0.081	4.50	9.04
4*	San Dimas - Chino Hills	22	0.686	0.722	0.043	0.078	4.60	6.93
5	Redlands	5	0.653	0.654	−0.006	0.078	4.37	4.37
6	Northwestern Riverside	11	0.733	0.750	−0.023	0.034	4.73	11.43
7	Southwestern Riverside	12	0.784	0.759	−0.034	0.010	5	10.82
8	Santa Ana Mountains	27	0.733	0.743	0.020	0.049	4.79	19.43
9*	San Joaquin Hills	36	0.751	0.758	0.008	0.033	4.70	22.67
10	South Camp Pendleton	35	0.746	0.779	0.047	−0.006	4.97	32.00
11	Northwest San Diego	11	0.766	0.764	−0.005	−0.006	4.86	21.32
12	San Pasqual	10	0.692	0.733	0.073	0.031	4.71	10.32
13	Cardiff - Los Penasquitos	16	0.712	0.742	0.033	0.039	4.89	14.04
14	Mission Trails	6	0.596	0.681	0.129	0.061	4.52	16.07
15	Lakeside	6	0.733	0.720	−0.034	0.018	4.66	12.46
16	San Diego City	8	0.731	0.741	0.012	0.014	4.81	18.00
17	Sweetwater	16	0.742	0.758	0.020	0.022	4.95	13.59
18	Otay - Jamul	22	0.733	0.771	0.050	0.002	5.02	15.99

Estimates are based on the full dataset and do not exclude close relatives. Asterisks denote regional aggregations with significantly different allele frequencies based on exact tests for population differentiaton in analyses including relatives. All other aggregations grouped together into one southern population. Effective population size estimates (N_e_) are included for the entire genetic cluster, estimated with the linkage disequilibrium method^71^ (top) and with the Colony full likelihood method^57^ (bottom).

**Figure 3:**
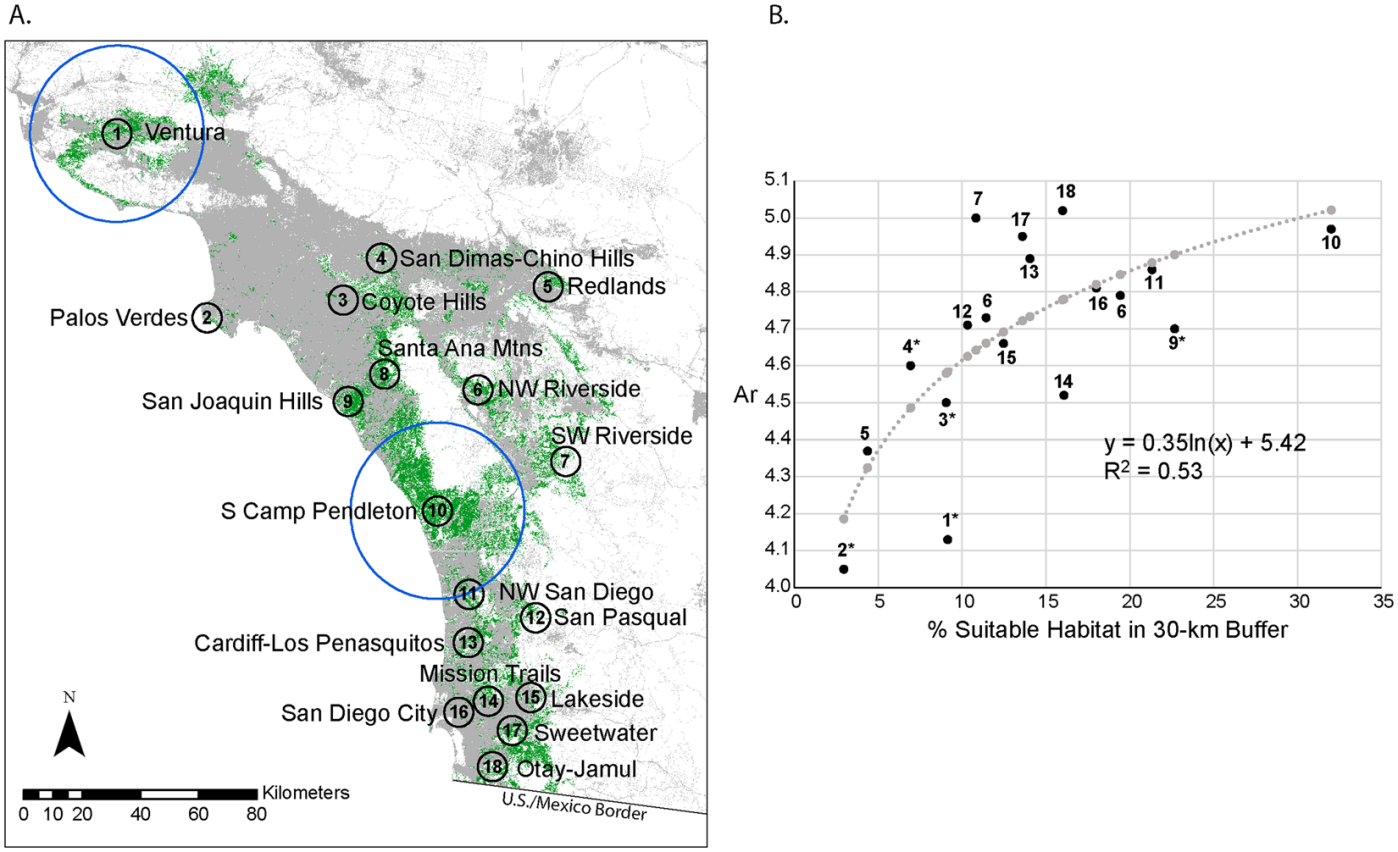
(**A**) Map of mean center points for 18 regional aggregations overlaid on suitable habitat (green). Black circles represent 5-km buffers. Two examples of 30-km buffers are shown as blue circles around Ventura and South Camp Pendleton. Ocean area was excluded from calculations of suitable habitat availability. (**B**) Relationship between allelic richness (*A*_r_) and percent suitable habitat of total land area within 30-km buffers. Data points are shown in black with aggregation numbers as in Panel A and Table 2. Model predictions are shown in grey. We found a statistically significant positive logarithmic relationship.

Overall, the California gnatcatcher habitat model performed well with a median HSI of 0.69 for the calibration dataset (n = 1,063) and 0.64 for the validation dataset (n = 3,225). We hypothesized that within aggregation genetic diversity would be positively correlated with availability of suitable habitat if suitable habitat was associated with regional population sizes and connectivity. Given that the set of aggregated sampling locations was small (n = 18), we constructed and evaluated simple linear and non-linear models relating genetic diversity to environmental variables, including available suitable habitat within 5-km and 30-km surrounding aggregations, which we calculated from the habitat model. Measures of genetic diversity including allelic richness (*A*_r_), relatedness (r) and expected heterozygosity (*H*_e_) were all highly correlated (r > 0.8). We selected *A*_r_ to represent genetic diversity in the modeling. Moran’s I measure of spatial autocorrelation was insignificant (p > 0.05) and we dropped latitude and longitude from our models. The number of samples from a sampling aggregation was not included in the models as it was highly correlated with the availability of suitable habitat within 5-km and 30-km buffers around the midpoint of sampling aggregations. There was no association between genetic diversity and the availability of suitable habitat at the 5-km scale. At the 30-km scale, however, availability of suitable habitat was positively related with *A*_r_ and best described by a logarithmic function that accounted for 53% of the variation in *A*_r_ (Fig. 3). Genetic diversity declined steeply when suitable habitat within 30 km of aggregations fell below 10 percent.

## Discussion

### Distinguishing recent connectivity from remaining signature of high historical connectivity

Conservation applications of population genetic data often focus on determining the impacts of anthropogenic changes to habitat availability and spatial connectivity on species genetic structure and diversity. However, both recent and historical population processes are reflected in a species’ genetic structure^36^. When there is no detectable population structure and underlying habitat is not contiguous, the pattern could support one of two hypotheses: first, that ongoing dispersal and gene flow among habitat patches are sufficient to retain a single genetic population; second, that signatures of high historical connectivity are retained despite recent loss of movement in response to habitat fragmentation. Here we further investigated recent dispersal distances from genetic pedigree analysis. About 25% of our sampled birds were identified in putative first order relative pairs and 38% of 34 relative pairs included individuals sampled in different regional aggregations, suggesting that there is recent or ongoing dispersal contributing to genetic similarity across the study area. Even at low frequencies, long distance dispersal can impact gene flow and genetic structure^8,37^. Analyses to identify close relatives are often performed in population genetic studies. Results are typically used to exclude relatives from population-level analyses to avoid confounding population-level and family structure^38^ (although this strategy is not always recommended^34^). Less often, however, is the spatial distribution of these relatives examined to provide additional information on the scale of recent dispersal. As our example indicates, it can be useful in this context.

### Connectivity in the coastal California gnatcatcher

Whether the gnatcatcher is a fragmentation-sensitive species has been the subject of some debate. Previous species richness studies in southern California found that gnatcatchers were absent from very small fragments in urban areas^39^. However, others have suggested that gnatcatchers do disperse through urban areas^40^, and a more recent regional analysis of gnatcatcher occupancy found no relationship between occupancy and patch size^41^. We recovered genetic signatures that are consistent with high connectivity and gene flow among most aggregations of gnatcatchers. Additional information from the distribution of first order relatives further supports very recent or ongoing gene flow in gnatcatchers across disparate aggregations. These findings suggest that gnatcatchers are able to move among most patches of suitable habitat given the current (or recent) spatial arrangement of coastal sage scrub habitat in southern California and their intrinsic movement and dispersal behavior. The possible exceptions are the outlying aggregations in more northern and isolated areas including Ventura, Palos Verdes, Chino Hills, Coyote Hills, and San Joaquin Hills. Relative pairs detected within three of these five aggregations (Ventura, Palos Verdes and Coyote Hills) were exclusively found within these same aggregations and none shared with other locations, further supporting greater isolation of these locations. There is more urban development and less suitable habitat in the northern portion of the range, which may contribute to the population structure detected there. With the exception of Ventura, signals of differentiation among northern aggregations were lost when close relatives were removed from the dataset; however, this may reflect the reduced sample size and lower statistical power in these reduced datasets rather than non-random sampling in these relatively small aggregations^34^. The stronger signal of differentiation between Ventura and other sites may reflect both distance and isolation. There appears to be very little suitable habitat connecting Ventura to more southern aggregations based on the habitat model (Fig. 1). However, further surveys of small patches of habitat in the Los Angeles Basin could help determine whether these sites are occupied and contribute to regional connectivity.

An important inference from our data is that gnatcatchers may be able to disperse farther than previously estimated. Previous resighting studies of juvenile dispersal distances in gnatcatchers reported most individuals resighted less than 4 km from their natal sites with maximum observed distances of 14 km^42^. Our median distance estimate among relatives is of similar scale (median Euclidean distance = 4.9 km, Fig. 2). Some of our relative pairs were detected much farther apart (Fig. 2, Table 1), although distances between relatives could overestimate dispersal. For example, recorded distances among relatives could be up to twice as far as actual dispersal distances if individuals dispersed from a central point, or could also represent multiple shorter movement steps over time rather than a single long dispersal event. On the other hand, resighting studies will underestimate dispersal distance, particularly when the spatial scale of the study is limited^6^. Our current genetic study extent far exceeds the extent of these previously reported mark-recapture efforts^40,42^. The study design of these previous efforts was not adequate nor likely intended to capture rare long-distance dispersal events.

Our results suggest recolonization of previously occupied habitat in recently disturbed areas is possible and may depend more on local habitat quality and less on dispersal limitations. However, further direct study of occupancy and recolonization rates after wildfire and other major disturbances is needed. Regional connectivity is likely dependent on local conditions and individual interactions as well as the availability of habitat between populations and thus may fluctuate over time or with further habitat modification.

### Patterns of genetic diversity

The positive relationship between suitable habitat and genetic diversity at the broad (30-km) scale suggests that areas with more suitable habitat likely have larger and more well-connected populations than those with less suitable habitat. While California gnatcatchers move through human modified landscapes, there appears to be a threshold in loss of suitable habitat where diversity within aggregations drops and population structure begins to develop. This was most evident in the northern part of the range where urbanization is highest and the amount of suitable habitat is lower. Lower diversity and greater isolation in the northern part of the study area may have importance as this is also the northern range edge for the species. Patterns of lower diversity at range edges are common among species and expected when habitat is not contiguous^43,44^. The “leading” range edge population in Ventura, for example, may be particularly important in allowing for future range shifts in response to climate change^45^. There are some recent extralimital observations and newly detected small populations of gnatcatchers north of the historical range^45^. However, lower connectivity with the main portion of the range due to both distance and the lack of intervening suitable habitat may also make the Ventura aggregation more vulnerable to local extirpation, particularly following major habitat disturbances from several recent large fires and drought.

Current recommended thresholds for effective population sizes are greater than 100 to avoid inbreeding depression in the short term and greater than 1000 to retain adaptive potential in the long term^46,47^. The total population of gnatcatchers exceeds the lower threshold, and may meet or exceed the upper threshold. This may bode well for continued long-term persistence of gnatcatchers in southern California. However, should habitat become further reduced and fragmented or local aggregations fluctuate dramatically in size, we could also see concomitant reductions in genetic connectivity and local genetic diversity over time. Such changes could be detected by regional population monitoring, which could benefit from coordinated efforts across San Diego, Orange, Western Riverside, San Bernardino and Los Angeles Counties, as birds in these regions form a cohesive genetic unit.

## Conclusions

We found that investigating the distribution of first order relatives was a useful strategy to help distinguish between recent gene flow and historical connectivity signals in population genetic data. This approach indicated that dispersal in California gnatcatchers may be less limited than previously estimated from banding studies. From a conservation perspective, it is encouraging that gnatcatchers retain genetic similarity and a large effective population size across the majority of their range in southern California. High estimated genetic connectivity and large effective population size are positive indicators that gnatcatchers could persist in southern California under current conservation and management strategies, which rely on a network of preserves. The exceptions appear to be more isolated northern aggregations where suitable habitat surrounding aggregations and genetic diversity within aggregations are lowest. Overall, recent habitat fragmentation seems to have had less impact on genetic connectivity in gnatcatchers than observed in a co-occurring songbird, the coastal cactus wren, (*Campylorhyncus brunneicapillus sandiegensis*), which has declined dramatically in abundance and has been impacted by genetic isolation within the same preserve network^4^. The cactus wren has lower maximum observed dispersal distances (up to 10 km), and requires mature cactus stands for nesting, which are patchy within coastal sage scrub habitat. This greater habitat specificity could contribute to greater observed population structure.

The coastal California gnatcatcher’s conservation status as a flagship species has assisted in the preservation of sage scrub habitat in southern California over the past several decades, which in turn provides habitat for numerous other sage scrub-dependent species. Given the contrasts in genetic connectivity between gnatcatchers and coastal cactus wrens over the same area, however, the gnatcatcher may be a poor monitoring surrogate to estimate persistence in other, more fragmentation-sensitive species. Finally, although current habitat conditions appear to support gnatcatcher connectivity in southern California, further loss or degradation of habitat could lead to future loss of connectivity, as seen in the emerging population structure and lower genetic diversity apparent within northern aggregations.

## Methods

### Field sampling and extractions

We sampled 268 gnatcatchers between May 2012 and September 2013. Sampling locations were chosen based on previous knowledge of presence from regional monitoring activities to provide a geographically representative sample of their U.S. range (Fig. 1). Gnatcatchers were captured in mist nets using song playbacks to attract birds to nets. All captured individuals were weighed, sexed, aged and banded with U.S. federal bird bands. We obtained blood via toenail-clipping or pulled growing feathers from each individual. All birds were handled in accordance with protocols approved by the U.S. Geological Survey, Western Ecological Research Center and U.C. Davis Institutional Animal Care and Use Committee (IACUC) and permitted under Master Banding Permit 22372, federal permit TE-829554-17 and a Memorandum of Understanding with the California Department of Fish and Wildlife held by BEK.

Samples were stored in either Queen’s lysis buffer with SDS or Qiagen ATL lysis buffer at −20 °C. In addition, two muscle tissue samples (frozen) were provided by the San Diego State University Museum of Biodiversity. These muscle tissues provided sufficient quantities of genomic DNA for microsatellite library development. All extractions were performed with the DNA Tissue Extraction Kit (Qiagen), each with 20 μL of dithiothreitol added for a digestion step extended to 48 hours. Extracted samples were quantified on a Nanodrop spectrophotometer (Thermo Scientific) and diluted to a maximum of 50 ng/μL prior to PCR amplification.

### Microsatellite Library Development

A shotgun library from one gnatcatcher was prepared using a Roche 454 Jr, providing 4,336 sequences with microsatellites. Of these, we screened 66 for variation using a three-primer technique^48^, and we found 22 of the loci were variable. We also tested loci from previously-developed libraries, finding successful cross-amplification of three loci originally developed for the cactus wren (*Campylorhynchus brunneicapillus sandiegensis*)^4^ and one locus for the southwestern willow flycatcher (*Empidonax traillii extimus*)^49^. Samples were genotyped on an ABI 3730 DNA Analyzer using the GS600 size standard (Life Technologies) at Bio Applied Technologies Joint, Inc. in San Diego, CA.

### Data Quality

We amplified 26 variable loci in three sets using the standard conditions of the Multiplex PCR Kit (Qiagen) with loci combined as indicated in Supplementary Table S1. Approximately 10% of samples were amplified and genotyped twice to confirm genotypes and estimate an error rate. Loci were checked for stepwise mutation model (SMM) consistency (including stutter and presence of null alleles) using MICRO-CHECKER^50^, and exact tests for Hardy-Weinberg equilibrium (HWE) and linkage disequilibrium (LD) among loci were performed in GENEPOP^51^. Loci with inconsistent amplification or that did not consistently conform to HWE and LD expectations were eliminated from the dataset. Individual genotypes and location data are available as a U.S. Geological Survey Data Release 10.5066/F77D2SBP.

### Identifying Populations and testing for genetic structure

We used a suite of individual clustering methods and exact tests for population genetic differentiation to determine genetic structure across the range. We used Bayesian clustering algorithms implemented in GENELAND^52^ and STRUCTURE^53^ to determine the number of gene pools. Both programs use genotypic data to assign individuals to clusters that conform to theoretical expectations for randomly mating populations (maximizing Hardy-Weinberg equilibrium and linkage equilibrium). GENELAND takes geographic relationships into consideration along with individual genotypic data, and can identify recently developed clusters^54^. Analyses were conducted using the uncorrelated alleles model with admixture, testing for clusters (*K*) between 1 and 10 with 1 million Markov chain Monte Carlo repetitions (MCMC) and a 20% burnin period. We followed the authors’ recommendations to use the uncorrelated alleles model in cases where isolation by distance is detected^52^. We explored genetic structure without geographic priors included in STRUCTURE^53^. We used the correlated alleles model with admixture for *K*s between 1 and 10, with a burnin period of 100,000 MCMC steps followed by 1,000,000 additional steps, and 20 repetitions at each *K*. The top 10 highest likelihood runs at each *K* were analyzed using STRUCTURE HARVESTER^55^, which averages and graphs likelihoods across runs. We used CLUMPP^56^ to average results across runs, and these results were visualized in DISTRUCT^57^. We also employed an individual, centered principal components analysis (PCA) in the R package Adegenet^58^ to summarize patterns of genetic diversity among individuals without imposing a population genetic model. We plotted individuals by the first four principal axes to visualize patterns of differentiation (data shown for first 2 axes).

As an additional means of defining genetically differentiated populations we used the method of Waples and Gaggiotti^33^ which uses exact tests to determine significant differences in allele frequencies among putative populations. If no difference is found, populations are grouped. This method can be more sensitive at detecting recently formed or weaker levels of genetic structure than clustering algorithms^33^. We used 18 local aggregations to begin this analysis. These aggregations were defined as local groupings of 5 or more individuals within a maximum distance of 30 km and not separated by urban development. We chose to use a maximum distance of 30 km as it is approximately twice the maximum dispersal distance reported in mark recapture studies^42^ and the average distance found between first order relative pairs in this study (see results), and so could represent a contact distance between individuals that is relevant for population structuring. Because we were interested in capturing potential impacts of habitat fragmentation, if groups of individuals were separated by expanses of urban development, we retained these as separate aggregations even if the distance among them was less than 30 km. We conducted exact contingency tests for pairwise genetic differentiation between all aggregations in GENEPOP, using Fisher’s method to combine p-values across loci^33^. To limit the effects of individual loci on the overall test, *p*-values for individual loci were set to a minimum of 0.0001 prior to combining with Fisher’s method. Aggregations were assumed to be part of the same genetic population if the overall *p*-value for the exact test was greater than 0.01. We did not use an adjustment for multiple tests as this adjusted *p*-value is already restrictive. We also calculated pairwise F_ST_ among all aggregations in GenAlEx 6.50, and tested for an association between pairwise genetic and geographic distances using a Mantel test for matrix correlation in the program IBD 1.53.

Because close relatives might be a source of bias in analyses of population genetic structure^38^, we repeated clustering and genetic differentiation analyses using reduced datasets with one of each first order relative pairs removed (see below for sibship assignment methods). The removal of close relatives, however, has also been shown to reduce the precision of genetic structure analyses^34^. Hence, we report observed differences in these results between analyses on these two datasets.

### Inferred Dispersal Distances

To estimate individual relationships and dispersal distances, we examined the spatial arrangement of genetically detected first order relatives (full siblings or parents and offspring). We used the program COLONY 2.0.6.2^24^ to identify putative sibship and parent offspring pairs within our dataset based on genetic similarity. For COLONY runs, we assumed an outbreeding model and a monogamous mating system, and coded all individuals as potential offspring. Known males and known females (based on field assignments) were coded as potential fathers and mothers respectively. Based on recent regional population size estimates of 1000–2000 pairs^41^, we set sampling percentages of parents at 5%. We applied the weak sibship prior, full likelihood model with the ‘very long run’ option (1 × 10^9^ iterations). To examine stationarity, we conducted three independent runs with unique random number generator seeds and compared results.

To assess accuracy in sibship assignments, we used the simulation module in COLONY to generate and analyze five replicate datasets using empirical allele frequencies and identical run conditions to our dataset. We set population size to 2000 with a 10% sampling rate (full run parameters are found in Supplementary Table S4). We examined the proportions of true vs. inferred fullsib, nonsib, parentage and non-parentage pairs summed across replicates.

We mapped the capture locations of well-supported putative first order relative pairs (prob > 0.90; recovered in all runs) and measured the Euclidean and least cost path distances between them to estimate the scale of recent dispersal. Least cost path distances were calculated in ArcGIS 10.2.2 from a friction surface with values calculated as the inverse of a gnatcatcher habitat suitability model (see modeling methods below). We also noted whether relatives were found within the same or among different aggregations.

### Landscape variables and habitat suitability modelling

We used gnatcatcher occurrences and an environmental dataset for southern California to develop a partitioned Mahalanobis D^2^ model predicting suitable habitat for gnatcatchers^59,60^. Mahalanobis D^2^ measures the similarity between points in a modeled landscape and the multivariate mean for a set of environmental variables calculated at locations where the species is present^61^. The environmental data set consisted of a grid of climatic, topographic, and vegetation/land use variables for southern California. We used Geographic Information Systems (GIS) software (ESRI 2013) to calculate environmental variables at each point in a grid of points spaced 150 m apart (Supplementary Table S5). To calibrate the models, we assembled 1,063 spatially distinct and precise California gnatcatcher observations collected between 1998 and 2013 from this study and others^62–66^. Observations had spatial accuracy of ≤160 m (the majority being more precise GPS coordinates) and only one location per 150-m × 150-m grid cell was used. We used gnatcatcher records compiled by the Carlsbad U.S. Fish and Wildlife Service Office^67^ to validate the models. The validation dataset included 3,225 spatially distinct gnatcatcher locations collected between 2000 and 2013.

We modified SAS 9.4^68^ code developed for previous modeling efforts^60,69^ to develop a partitioned Mahalanobis D^2^ habitat model for the gnatcatcher that included spatial subsampling and model averaging. To reduce biasing the model towards environmental conditions at locations with relatively large concentrations of gnatcatchers, we divided the range into six geographic areas: LA-Ventura; Orange; Riverside-San Bernardino; Western Riverside; San Diego Coastal; and San Diego Inland. We bootstrapped the calibration data by limiting each geographic area to 50 or fewer occurrences per iteration and performed a Principal Components Analysis (PCA) on each of 1000 samples^69^. We produced a final model by averaging the PCA output for all iterations after correcting for sign ambiguity^69,70^. We rescaled D^2^ from 0–1 to create a continuous habitat similarity index (HSI) and calculated an HSI value for each 150-m × 150-m grid cell. An HSI of “0” represents environmental conditions that are least similar to the multivariate mean for the species occurrence dataset used to calibrate the model (i.e., unsuitable), while a “1” represents those conditions that are most similar (i.e., suitable). We applied a threshold of 0.5 to define high and very high suitable habitat to calculate least cost dispersal paths and availability of suitable habitat within aggregations (see below). This HSI threshold is consistent with high and very high suitability classifications that were found to be positively associated with gnatcatcher occupancy in previous modeling efforts^41,71^.

### Genetic Diversity Patterns

We examined genetic diversity indices within 18 regional aggregations of gnatcatchers to determine how these might vary across the distribution and with local habitat availability. For regional aggregations, we calculated observed (*H*_o_) and expected (*H*_e_) heterozygosity and the fixation index in GenAlEx 6.501^72,73^, and allelic richness (*A*_r_), adjusted for sample size, in HP-RARE^74^. Average pairwise relatedness among individuals within aggregations (r)^35^ was calculated in GenAlEx. We examined relationships between these diversity indices and geographic location (latitude and longitude), sample size within aggregations (N) and percent suitable habitat surrounding aggregations. To calculate percent suitable habitat, we first mapped the mean center point of each of the 18 defined local aggregations. We extracted the amount of suitable habitat relative to the total land cover in 5-km and 30-km radius buffers around each of these points, and expressed this as percent suitable habitat. These radii were chosen to correspond to the median and mean dispersal distances that we inferred from distances among relatives. We used these dispersal distance estimates to determine areas that could include groups of interacting and potentially interbreeding individuals (populations).We ran correlation tests among the four genetic diversity indices, and for those that were highly correlated (r > 0.7) we selected one diversity index for modeling. Using R software^75^ we ran correlation tests between independent variables and retained the most biologically relevant variable in the model when two variables were highly correlated (r > 0.7). We used Moran’s I in ArcGIS 10.5 software to test for spatial autocorrelation between genetic diversity measures and environmental correlates. Simple linear and non-linear models of genetic diversity were fit with uncorrelated independent variables. We evaluated model fit based on scatterplots, residual plots, R^2^, and residual standard errors.

We estimated the effective population sizes (N_e_) for the total population supported by clustering methods. Effective population size was estimated in the program NeESTIMATOR 2.01^76^, using the linkage disequilibrium method, assuming random mating and using a minimum allele frequency of 0.01. Confidence intervals (95%) were obtained by jackknifing over loci. A second estimate of N_e_ was obtained from sibship assignments using the full likelihood method in COLONY^24^, assuming random mating, with 95% confidence estimates obtained from bootstrapping.

## Supplementary information

Supplementary Information
