# Supplementary material for: Distinguishing recent dispersal from historical genetic connectivity in the coastal California gnatcatcher
Source: Sci Rep. 2019 Feb 4;9:1355. doi: 10.1038/s41598-018-37712-2 (PMC6362141; doi:10.1038/s41598-018-37712-2)

**Distinguishing recent dispersal from historical genetic connectivity in the coastal California gnatcatcher.**

**Supplementary Information.**

Amy G. Vandergast^1^*, Barbara E. Kus^1^, Kristine L. Preston^1,2^, Kelly R. Barr^1,3^

^1^ U. S. Geological Survey, Western Ecological Research Center

^2^ San Diego Management and Monitoring Program

^3^ Current affiliation: Center for Tropical Research, Institute for the Environment and Sustainability, University of California-Los Angeles, Los Angeles, CA 90095

**Supplementary Table S1:** Information about microsatellite loci retained in analyses, including the length and motif from the original sequence, putative chromosome location (Chr), multiplex mix membership (MP), and primer sequences (5’ to 3’).

| Locus | Length | Motif | Chr | MP | Forward Primer | Reverse Primer |
| --- | --- | --- | --- | --- | --- | --- |
| CAGN4-01 | 160 | (GTAT)^6^GAATCTG(TCTA)^11^ | 1 | 1 | TTTTGCCTAATAAACTGGCTGAC | GTTTCTTCACAGAACCACAACCTACATGG |
| CAGN03-13 | 262 | (ACT)^7^ | 3 | 1 | AACAGTGACATACAAGAATTCAGC | GTTTCTTCAGAAACTCACAGCCAGCAC |
| CAGN3-15 | 250 | (ATC)^15^ | 5 | 1 | TCTCCTTGGTTAGGATGCAAG | GTTTCTTCTGGATGATGATGCTTGCTG |
| CAGN3-35 | 180 | (TAT)13 | -- | 1 | TTGTCTATCATTGGTCACATACCC | GTTTCTTGCACAGAGGGATTTCACAGG |
| CAGN3-36 | 291 | (ATT)^13^ | -- | 1 | ACAGCTCCTGGAGGAGAGAG | GTTTCTTCAAACCCTGTTTGTTAATAGTG |
| CAGN3-45 | 203 | (TTA)^8^ | 4A | 1 | AATCTTCTGTGGTGCCATCC | GTTTCTTAGGCCTGAGTCCGTAGCAC |
| CAGN4-10 | 129 | (AAT)^9^ | -- | 1 | CCGAGAGATGGACAATCCAC | GTTTCTTGGGTGCAGAGACACAAGGAG |
| CAGN5-03 | 188 | (GCACA)^9^ | -- | 1 | AAGAAGGAGCGGAGGACATC | GTTTCTTACGGAGGCTACACACTGCTC |
| CAGN5-07 | 263 | (ATTGG)^11^ | -- | 1 | GGTTGGGTTAGACTGAATTGG | GTTTCTTACCAGGTGTGAGCAGCAAC |
| CAGN5-09 | 319 | (AGAAT)^11^ | -- | 1 | CACCCATTCTTGTTTGATCC | GTTTCTTCAGTGATAGGAGGCATTTGG |
| CAGN03-12 | 302 | (GCT)^6^ | 3 | 2 | GTTTGGCGAAGAGCAGGTAG | GTTTCTTCAGGCATATTGCCTTTGAGG |
| CAGN04-02 | 198 | (ACAG)^10^ | 9 | 2 | ATCCTGCTCGAACAATCAGC | GTTTCTTACGGCCAAAGTGAGTACGG |
| CAGN3-39 | 236 | (AGG)^10^ | -- | 2 | ATGCCATCACTTCCCAAATC | GTTTCTTGCACTCAGCAAACAATTCAC |
| CAGN3-41 | 129 | (TGAT)^8^ | -- | 2 | TGAAGTCAGTGTTGAGGACCAG | GTTTCTTCATAAGCTTGACTAGATTCTCTGC |
| CAGN4-09 | 90 | (CAAT)^9^ | -- | 2 | CCCATCCTGCTGTGTGTG | GTTTCTTCTGGCACAAAGTTTGCACTAAAG |
| CAGN5-02 | 228 | (CAGAG)^10^ | -- | 2 | AACAGGTCTGTGTCCTTCCTG | GTTTCTTAGAACTTGGTGGTGCTGGAC |
| CAGN5-06 | 160 | (ATAAC)^13^ | -- | 2 | TTTGGGAGGTATGGGATGC | GTTTCTTACCTGCAAGCAAGAAAGCAC |
| CAGN5-08 | 205 | (AATGG)^10^ | 4 | 2 | TGAATTTGATCCAGGGCAAG | GTTTCTTGCTATTCCCTCAGTACAGCAATG |
| CAGN6-02 | 263 | (TTATTC)^13^ | -- | 2 | TCCTGCAATGTCAAAGTGTTG | GTTTCTTCAATTACAATGGAATCACAAACTG |

**Supplementary Table S2:** Population differentiation among six genetically distinguishable populations, defined using sequential Exact Tests, following Waples and Gaggiotti (2006). Fisher’s combined probability test Χ^2^ values are presented below the diagonal and associated *p*-values are above the diagonal, with degrees freedom on diagonal. The southern population is a combination of 13 aggregations.

|  | **Ventura** | **Palos Verdes** | **San Dimas-Chino Hills** | **Coyote Hills** | **San Joaquin Hills** | **So. Population** |
| --- | --- | --- | --- | --- | --- | --- |
| **Ventura** | 38 | <0.0001 | <0.0001 | <0.0001 | <0.0001 | <0.0001 |
| **Palos Verdes** | 134.64 | 38 | <0.0001 | 0.002 | <0.0001 | <0.0001 |
| **San Dimas-Chino Hills** | 140.04 | 101.75 | 38 | 0.0002 | <0.0001 | <0.0001 |
| **Coyote Hills** | 140.14 | 66.68 | 76.39 | 38 | <0.0001 | <0.0001 |
| **San Joaquin Hills** | 143.12 | 99.83 | 135.76 | 105.8 | 38 | <0.0001 |
| **Southern Population** | 156.09 | 95.87 | 139.75 | 110.92 | 125.98 | 38 |

**Supplementary Table S3:** F_ST_ ^1^ between all pairs of aggregations (below diagonal) and associated *p-*values based on 9999 permutations (above the diagonal).

|  | **1** | **2** | **3** | **4** | **5** | **6** | **7** | **8** | **9** | **10** | **11** | **12** | **13** | **14** | **15** | **16** | **17** | **18** |
| --- | --- | --- | --- | --- | --- | --- | --- | --- | --- | --- | --- | --- | --- | --- | --- | --- | --- | --- |
| **1** |  | 0.000 | 0.000 | 0.000 | 0.005 | 0.001 | 0.001 | 0.000 | 0.000 | 0.000 | 0.000 | 0.000 | 0.000 | 0.001 | 0.001 | 0.002 | 0.000 | 0.000 |
| **2** | 0.089 |  | 0.033 | 0.001 | 0.054 | 0.024 | 0.003 | 0.002 | 0.004 | 0.001 | 0.006 | 0.028 | 0.002 | 0.008 | 0.019 | 0.009 | 0.001 | 0.030 |
| **3** | 0.067 | 0.032 |  | 0.026 | 0.044 | 0.003 | 0.002 | 0.025 | 0.001 | 0.002 | 0.000 | 0.001 | 0.004 | 0.000 | 0.007 | 0.008 | 0.001 | 0.000 |
| **4** | 0.050 | 0.047 | 0.015 |  | 0.085 | 0.002 | 0.000 | 0.024 | 0.000 | 0.001 | 0.000 | 0.001 | 0.000 | 0.000 | 0.000 | 0.002 | 0.000 | 0.000 |
| **5** | 0.056 | 0.037 | 0.028 | 0.015 |  | 0.024 | 0.060 | 0.040 | 0.008 | 0.052 | 0.004 | 0.104 | 0.036 | 0.019 | 0.011 | 0.023 | 0.029 | 0.085 |
| **6** | 0.043 | 0.030 | 0.031 | 0.021 | 0.027 |  | 0.134 | 0.014 | 0.020 | 0.225 | 0.008 | 0.066 | 0.002 | 0.003 | 0.017 | 0.142 | 0.000 | 0.015 |
| **7** | 0.045 | 0.046 | 0.034 | 0.033 | 0.020 | 0.009 |  | 0.000 | 0.002 | 0.026 | 0.051 | 0.026 | 0.005 | 0.007 | 0.022 | 0.165 | 0.011 | 0.071 |
| **8** | 0.046 | 0.041 | 0.014 | 0.008 | 0.020 | 0.015 | 0.029 |  | 0.000 | 0.019 | 0.001 | 0.003 | 0.006 | 0.000 | 0.006 | 0.007 | 0.000 | 0.000 |
| **9** | 0.043 | 0.038 | 0.027 | 0.029 | 0.031 | 0.013 | 0.020 | 0.021 |  | 0.009 | 0.012 | 0.002 | 0.000 | 0.004 | 0.008 | 0.023 | 0.001 | 0.001 |
| **10** | 0.042 | 0.042 | 0.021 | 0.014 | 0.017 | 0.003 | 0.011 | 0.007 | 0.008 |  | 0.065 | 0.017 | 0.015 | 0.002 | 0.094 | 0.203 | 0.001 | 0.001 |
| **11** | 0.043 | 0.039 | 0.040 | 0.033 | 0.037 | 0.021 | 0.013 | 0.026 | 0.015 | 0.008 |  | 0.141 | 0.200 | 0.157 | 0.193 | 0.477 | 0.095 | 0.393 |
| **12** | 0.049 | 0.029 | 0.036 | 0.024 | 0.016 | 0.013 | 0.017 | 0.021 | 0.024 | 0.013 | 0.008 |  | 0.168 | 0.012 | 0.018 | 0.159 | 0.007 | 0.300 |
| **13** | 0.046 | 0.044 | 0.024 | 0.022 | 0.022 | 0.024 | 0.021 | 0.014 | 0.022 | 0.010 | 0.005 | 0.007 |  | 0.006 | 0.048 | 0.184 | 0.039 | 0.030 |
| **14** | 0.058 | 0.054 | 0.064 | 0.065 | 0.043 | 0.038 | 0.030 | 0.052 | 0.030 | 0.032 | 0.011 | 0.030 | 0.029 |  | 0.344 | 0.450 | 0.061 | 0.053 |
| **15** | 0.062 | 0.042 | 0.039 | 0.043 | 0.050 | 0.028 | 0.025 | 0.028 | 0.026 | 0.011 | 0.009 | 0.029 | 0.017 | 0.006 |  | 0.009 | 0.029 | 0.079 |
| **16** | 0.039 | 0.042 | 0.030 | 0.026 | 0.032 | 0.009 | 0.008 | 0.022 | 0.016 | 0.005 | 0.000 | 0.009 | 0.007 | 0.001 | 0.031 |  | 0.228 | 0.472 |
| **17** | 0.040 | 0.051 | 0.036 | 0.039 | 0.024 | 0.039 | 0.018 | 0.033 | 0.023 | 0.019 | 0.009 | 0.021 | 0.011 | 0.017 | 0.021 | 0.005 |  | 0.046 |
| **18** | 0.040 | 0.021 | 0.034 | 0.030 | 0.014 | 0.014 | 0.008 | 0.024 | 0.017 | 0.014 | 0.001 | 0.003 | 0.010 | 0.015 | 0.013 | 0.000 | 0.008 |  |

1 Weir, B. S. & Cockerham, C. C. Estimating F-statistics for the analysis of population structure. *Evolution* **38**, 1358-1370 (1984).

**Supplementary Table S4: Colony simulation full parameter settings.**

| **Parameter Setting** | **Value** |
| --- | --- |
| Number of replicates | 3 |
| 0/1/2=Pair-likelihood Score(PLS)/Full likelihood(FL)/FL-PLS combined (FPLS) method | 1 |
| 0/1/2/3 for low/medium/high/very high precision | 2 |
| 2/1=Dioecious/Monoecious | 2 |
| Number of mating structures | 100 |
| #dads & mums in a mating structure | 10 10 |
|  | 1 0 0 0 0 0 0 0 0 0 |
|  | 0 1 0 0 0 0 0 0 0 0 |
|  | 0 0 2 0 0 0 0 0 0 0 |
|  | 0 0 0 2 0 0 0 0 0 0 |
|  | 0 0 0 0 2 0 0 0 0 0 |
|  | 0 0 0 0 0 2 0 0 0 0 |
|  | 0 0 0 0 0 0 2 0 0 0 |
|  | 0 0 0 0 0 0 0 3 0 0 |
|  | 0 0 0 0 0 0 0 0 3 0 |
|  | 0 0 0 0 0 0 0 0 0 4 |
|  |  |
| #candidate males & females | 1000 1000 |
| Assumed prbs of fathers & mothers included in candidates | 0.1 0.07 |
| Number of Loci | 19 |
| Prob. of missing genotypes | 0 |
| Dropout rates | 0.001 0.001 0.001 0.001 0.001 0.001 0.001 0.001 0.001 0.001 0.001 0.001 0.001 0.001 0.001 0.001 0.001 0.001 0.001 |
| Other error rates | 0.001 0.001 0.001 0.001 0.001 0.001 0.001 0.001 0.001 0.001 0.001 0.001 0.001 0.001 0.001 0.001 0.001 0.001 0.001 |
| Codominant/Dominant (0/1) markers | 0 0 0 0 0 0 0 0 0 0 0 0 0 0 0 0 0 0 0 |
| #alleles/locus, must be=2 for dominant marker | 12 9 15 21 8 8 9 3 13 15 10 10 12 5 6 16 19 7 8 |
| 0/1/2/3=Uniform/Equal/Triangular/other allele freq. distr. | 3 |
|  | 0.088 0.011 0.021 0.007 0.278 0.274 0.09 0.022 0.091 0.075 0.009 0.034 |
|  | 0.032 0.116 0.237 0.175 0.174 0.091 0.147 0.004 0.024 |
|  | 0.06 0.229 0.08 0.007 0.091 0.134 0.127 0.022 0.118 0.017 0.017 0.062 0.017 0.017 0.002 |
|  | 0.022 0.013 0.026 0.013 0.049 0.002 0.071 0.004 0.101 0.002 0.159 0.2 0.082 0.155 0.019 0.024 0.021 0.022 0.007 0.004 0.002 |
|  | 0.125 0.09 0.541 0.024 0.052 0.049 0.118 0.002 |
|  | 0.024 0.091 0.386 0.216 0.071 0.11 0.063 0.037 |
|  | 0.116 0.037 0.274 0.065 0.366 0.037 0.086 0.007 0.011 |
|  | 0.528 0.213 0.259 |
|  | 0.011 0.017 0.038 0.047 0.364 0.104 0.218 0.034 0.042 0.051 0.028 0.036 0.009 |
|  | 0.142 0.06 0.036 0.114 0.043 0.006 0.004 0.217 0.002 0.006 0.242 0.086 0.036 0.004 0.004 |
|  | 0.032 0.015 0.093 0.312 0.272 0.006 0.194 0.013 0.042 0.021 |
|  | 0.028 0.4 0.006 0.468 0.002 0.004 0.017 0.051 0.023 0.002 |
|  | 0.032 0.036 0.085 0.214 0.132 0.158 0.135 0.064 0.086 0.021 0.028 0.009 |
|  | 0.198 0.502 0.188 0.101 0.011 |
|  | 0.248 0.14 0.33 0.131 0.146 0.006 |
|  | 0.134 0.153 0.004 0.035 0.132 0.004 0.052 0.082 0.09 0.073 0.084 0.075 0.043 0.026 0.011 0.002 |
|  | 0.006 0.036 0.117 0.117 0.024 0.083 0.094 0.081 0.038 0.098 0.055 0.053 0.041 0.039 0.034 0.036 0.008 0.03 0.013 |
|  | 0.12 0.044 0.019 0.148 0.283 0.112 0.274 |
|  | 0.038 0.142 0.015 0.01 0.046 0.329 0.052 0.367 |
| 1/n=HaploDiploid/n-ploid species | 2 |
| 1/0 =Mono/Polygamy for males & females | 1 1 |
| Seed for random number generator | 1234 |
| I,R,R : I=0,1,2,3 for No,weak,medium,strong sibship size prior R,R=mean paternal & maternal sibship size | 1 1 1 |
| B, 0/1=Clone inference =No/Yes | 0 |
| B, 0/1=Scale full sibship=No/Yes | 1 |
| 1/0 (Y/N) for known allele frequency | 1 |
| 1/0 for updating allele freq. or not | 0 |
| #replicate runs | 1 |
| 0/1/2/3/4=VeryShort/Short/Medium/Long/VeryLong run | 2 |
| Map length in Morgans. <0 for infinite length | -1 |
| Inbreeding coefficient of parents | 0.07 |
| 0/1=N/Y for allowing inbreeding in Colony | 0 |

**Supplementary Table S5:** Descriptions and sources of environmental variables included in habitat suitability modeling.

| **Variable** | **Description and Source** |
| --- | --- |
| Topographic variables: elevation (m), slope (degrees), northness (sine of aspect), eastness (cosine of aspect) | 10-m digital elevation model ^1,2^ |
| Topographic heterogeneity (ruggedness) | Calculated from the DEM (above) for 30m X 30m neighborhood of 10 m cells at each grid point ^3^ |
| Climate variables: minimum January temperature (°C), maximum July temperature (°C), February – May precipitation (mm) | Extracted at each grid point using normalized 30-year modeled estimates (1981-2010 ^4,5^) |
| Vegetation: Percent of landscape within 1 km of each grid point classified as coastal sage scrub, chaparral, and urban development | Fire Resource Assessment Program vegetation map ^6^ |

**References**

1 Gesch, D., *et al.* The national elevation dataset. *Photogrammetric Engineering and Remote Sensing* **68,** 5-11 (2002).

2 Gesch D.B. The National elevation dataset, *In: Digital Elevation Model Technologies and Applications: The DEM Users Manual*, 2nd Edition (ed, Maune, D) 99-118. (American Society for Photogrammetry and Remote Sensing, 2007).

3 Sappington, J. M., Longshore K. M., Thompson D. B. Quantifying landscape ruggedness for animal habitat analysis: a case study using bighorn sheep in the Mohave Desert. *Journal of Wildlife Management* **71**, 1419-1426 (2007).

4 Daly, C. *et al.* Physiographically-sensitive mapping of temperature and precipitation across the coterminous United States. *Journal of Climatology* **28**, 2031-2064 (2008).

5 PRISM. *PRISM climate GIS dataset*. PRISM Climate Group, Northwest Alliance for Computational Science and Engineering. <http://www.prism.oregonstate.edu/>. (2013).

6 Fire Resource Assessment Program, Department of Forestry and Fire Protection (FRAP) *Methods for Development of Habitat Data: Forest and Range 2002 Assessment.* Technical Working Paper 8-19-02. <http://frap.fire.ca.gov/projects/frap_veg/methods/Methods_Development_Habitat_Data_02_2.pdf>. (2002).

**Supplementary Table S6:** Cooperating organizations that provided property access and logistical support.

Audubon California Starr Ranch Sanctuary

Bureau of Land Management

California Department of Fish and Wildlife

California State Parks and Recreation

California State Polytechnic University, Pomona

California State University, Channel Islands

California Department of Transportation

Center for Natural Lands Management

City of Carlsbad

City of Chula Vista

City of Diamond Bar

City of El Cajon

City of Escondido

City of Fullerton

City of Glendora

City of Irvine

City of Moorpark

City of Oceanside

City of San Diego

City of San Dimas

City of Thousand Oaks

City of Whittier

Conejo Open Space Conservation Authority

Conejo Recreation and Parks District

Cooper Ecological Monitoring, Inc.

County of Los Angeles, Department of Parks and Recreation

County of Riverside, Economic Development Agency

County of San Diego

Crystal Cove State Park

Fallbrook Naval Weapons Station

Irvine Ranch Conservancy

Many Private Landowners

Marine Corps Base Camp Pendleton

Metropolitan Water District

Nature Reserve of Orange County

North Etiwanda Preserve

Orange County Water District

Orange County Parks

Palos Verdes Peninsula Land Conservancy

Puente Hills Habitat Preservation Authority

Riverside County Habitat Conservation Authority

Riverside County Parks

San Bernardino County Flood Control District

San Bernardino County Water Conservation District

San Bernardino Valley Municipal Water District

San Diego Monitoring and Management Program

San Diego National Wildlife Refuge

San Diego Zoo Institute for Conservation Research

San Dieguito River Park

San Dieguito River Valley Conservancy

Santa Ana Watershed Association

Sweetwater Authority

UC Irvine Ecological Preserve

US Fish and Wildlife Service

Western Riverside County MSHCP

Western Riverside County Regional Conservation Authority

Western Foundation for Vertebrate Zoology

**Supplementary Figure S1**: Results of Geneland Bayesian clustering analyses. Graph shows the number of clusters returned along the MCMC chain after burnin. A single cluster was most often recovered.


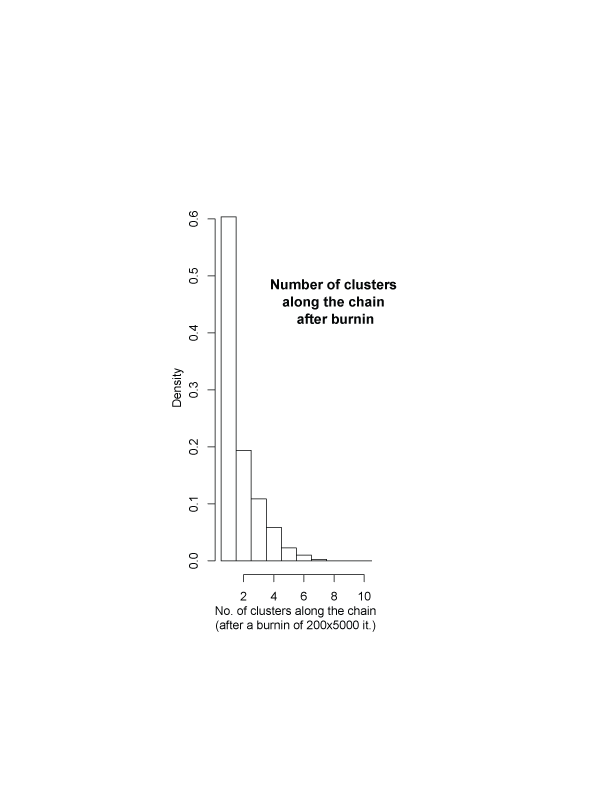


**Supplementary Figure S2. Results of Structure Bayesian clustering Analysis.** A) Plot of likelihood scores averaged across the top 10 runs at each K value. Highest likelihood scores were returned at K = 1, 2 or 3 clusters. B) Individual assignment plot for K of 2 arranged from North to South. Individuals of mixed assignment were found throughout the range and there was little association between geographic location and assignment. C) Individual assignment plot for K = 3 arranged from North to South. All individuals show mixed assignment with no geographic association.


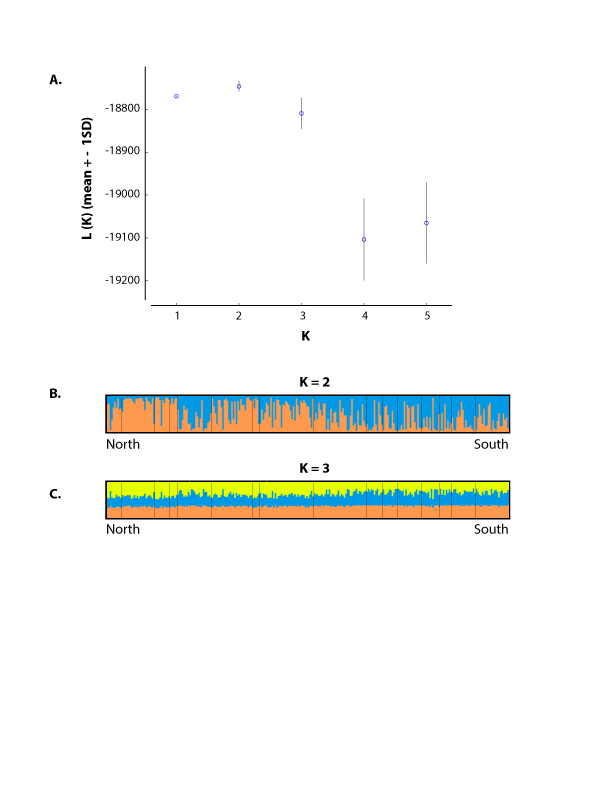


**Supplementary Figure S3:** Principal components analysis plot of PC axes 1 and 2 accounting for 49% of the total genetic variation. Individuals are grouped, colored, and labeled by 18 regional aggregations (see figure 2A for map of regional aggregations). Colored ellipses indicate the distribution of the individuals from different aggregations and show high overlap among aggregations. Inset shows the eigenvalue scree plot for the first 20 PC axes.


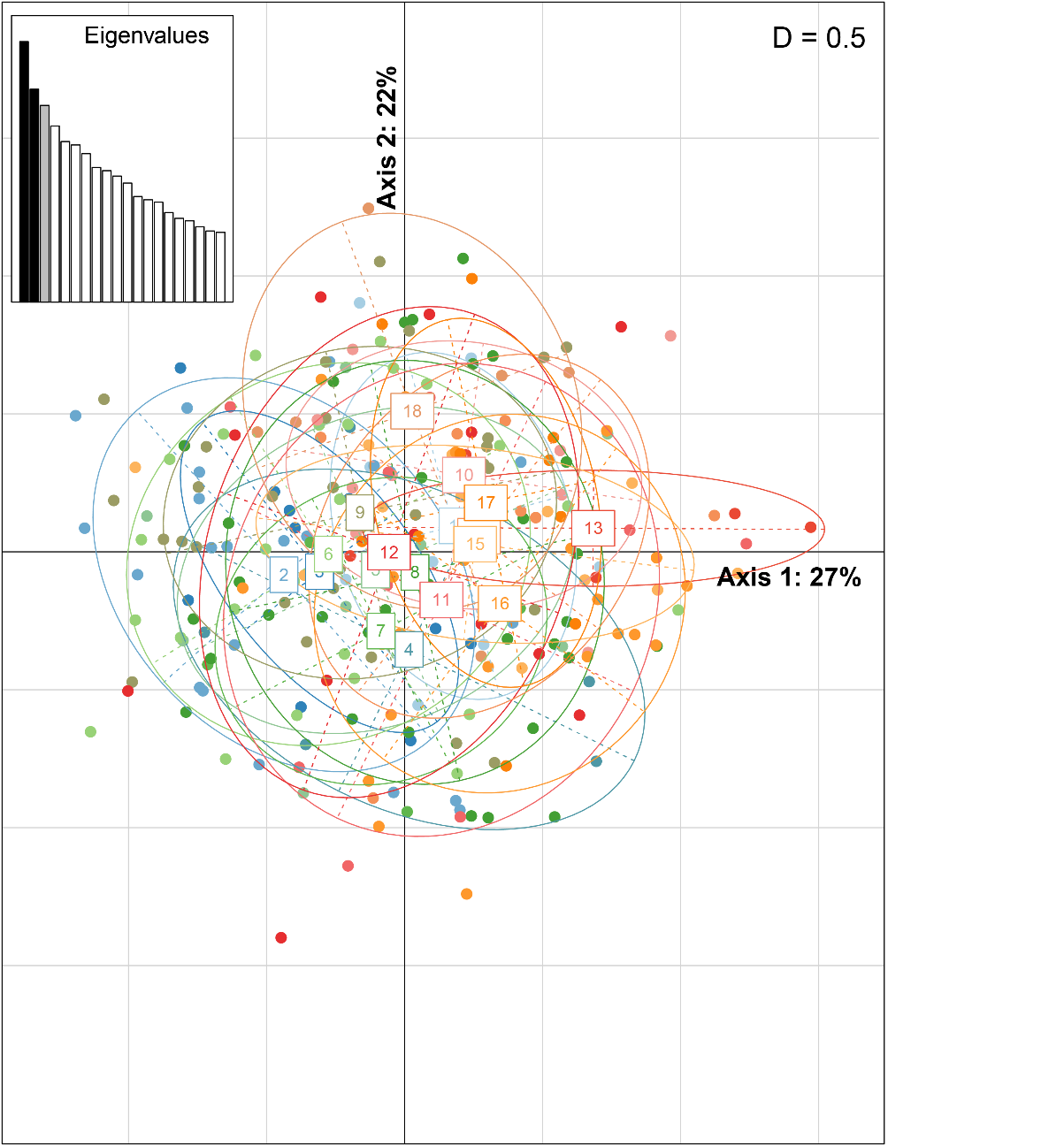

Supplement: Supplementary file 1 — Supplementary Information [file 41598_2018_37712_MOESM1_ESM.docx]
